# Functional Diversity of Transcriptional Regulators in the Cyanobacterium *Synechocystis* sp. PCC 6803

**DOI:** 10.3389/fmicb.2017.00280

**Published:** 2017-02-21

**Authors:** Mengliang Shi, Xiaoqing Zhang, Guangsheng Pei, Lei Chen, Weiwen Zhang

**Affiliations:** ^1^Laboratory of Synthetic Microbiology, School of Chemical Engineering and Technology, Tianjin UniversityTianjin, China; ^2^Key Laboratory of Systems Bioengineering – Ministry of Education, Tianjin UniversityTianjin, China; ^3^SynBio Research Platform, Collaborative Innovation Center of Chemical Science and EngineeringTianjin, China; ^4^Center for Biosafety Research and Strategy, Tianjin UniversityTianjin, China

**Keywords:** function, metabolomics, *Synechocystis*, transcriptional regulators, LC-MS

## Abstract

Functions of transcriptional regulators (TRs) are still poorly understood in the model cyanobacterium *Synechocystis* sp. PCC 6803. To address the issue, we constructed knockout mutants for 32 putative TR-encoding genes of *Synechocystis*, and comparatively analyzed their phenotypes under autotrophic growth condition and metabolic profiles using liquid chromatography-mass spectrometry-based metabolomics. The results showed that only four mutants of TR genes, *sll1872* (*lytR*), *slr0741* (*phoU*), *slr0395* (*ntcB*), and *slr1871* (*pirR*), showed differential growth patterns in BG11 medium when compared with the wild type; however, in spite of no growth difference observed for the remaining TR mutants, metabolomic profiling showed that they were different at the metabolite level, suggesting significant functional diversity of TRs in *Synechocystis*. In addition, an integrative metabolomic and gene families’ analysis of all TR mutants led to the identification of five pairs of TR genes that each shared close relationship in both gene families and metabolomic clustering trees, suggesting possible conserved functions of these TRs during evolution. Moreover, more than a dozen pairs of TR genes with different origin and evolution were found with similar metabolomic profiles, suggesting a possible functional convergence of the TRs during genome evolution. Finally, a protein–protein network analysis was performed to predict regulatory targets of TRs, allowing inference of possible regulatory gene targets for 4 out of five pairs of TRs. This study provided new insights into the regulatory functions and evolution of TR genes in *Synechocystis*.

## Introduction

Cyanobacteria contribute significantly to global photosynthetic productivity. It is estimated that more than half of the total primary production essential for life on earth is produced by cyanobacteria. In addition, early studies have found that cyanobacteria were able to establish competitive growth in almost any environment, at least temporarily, liquid water and sunlight, due to their strong abilities of withstanding challenges of environmental perturbations (Badger et al., 2006). Moreover, cyanobacteria have recently attracted significant interest because of their ability to function as a “*chassis*” to produce renewable carbon neutral biofuels or bioproducts (Atsumi et al., 2009). In spite of their important ecological, environmental, and biotechnological applications, many aspects of cyanobacterial physiology remain poorly understood.

To survive the diversities of environments, abundant and dedicated regulatory systems have evolved in cyanobacteria to achieve precise controls of functional gene expression. In the model cyanobacterium *Synechocystis* sp. PCC 6803 (hereafter *Synechocystis*), a significant number of regulatory genes of various types have been identified, among which at least 40 genes were annotated as putative transcriptional regulators (TRs) (Kaneko et al., 2003). So far only a dozen of TRs have ever been functionally characterized in *Synechocystis*, and the results showed that they were involved in the regulation of a wide range of physiological functions, such as nitrite tolerance (Aichi et al., 2001), iron limitation (Michel and Pistorius, 2004), acid tolerance (Ohta et al., 2005), cadmium tolerance (Houot et al., 2007). However, a majority of TRs in the *Synechocystis* genome are still functionally unknown, which presents significant challenges not only to the basic sciences of *Synechocystis* but also to the biotechnological application of *Synechocystis* as a chassis in producing biofuels and chemicals (Atsumi et al., 2009).

To decipher regulatory function of TRs, various approaches have been previously applied. For example, sequence analysis-based identification and evolutionary analysis of DNA-binding proteins, construction of transcriptional networks including TRs and their target genes and analysis of structure and evolution of these networks (Babu et al., 2004), can be used for functional inference of TRs. Metabolomics is a method to define the diversity of low weight molecules in the cell and to display differences in small molecule abundance. When applied for analysis of cellular responses to genetic or physiological changes, it shows many advantages because metabolites are the functional entities within the cells and their concentration levels vary as a consequence of environmental changes (Zhang et al., 2010). In our previous studies, metabolomic analysis has been applied to the functional characterization of response regulators involved in acid and butanol tolerance (Ren et al., 2014; Niu et al., 2015), and TRs involved in ethanol tolerance in *Synechocystis* (Zhu et al., 2015), and the results demonstrated that it could be a powerful tool in revealing functional clues for functionally unknown regulatory genes. Towards an ultimate goal of deciphering regulatory functions of TRs in *Synechocystis*, in this study, we applied a liquid chromatography-mass spectrometry (LC-MS) based metabolomics to a comparative analysis of knockout mutants for 32 putative *Synechocystis* TR-encoding genes (Zhu et al., 2015). The results showed significant functional diversity of TRs at the metabolic level in *Synechocystis*, as well as functional diversity of TRs based on their differential clustering patterns in relationship trees resulted from TR families and metabolomic clustering analysis. This study provided interesting information on the regulatory functions and evolution of TR genes in *Synechocystis*.

## Materials and Methods

### Bacterial Growth Conditions

*Synechocystis* sp. PCC 6803 was obtained from American Type Culture Collection (ATCC), and used as a wild type to construct single-gene knockout mutants of TR genes. A total of 32 knockout mutants of putative TR-coding genes were constructed, confirmed and described previously (Zhu et al., 2015). Briefly, for the gene target selected, three sets of primers were designed to amplify a linear DNA fragment containing the chloramphenicol resistance cassette (amplified from a plasmid pACYC184) with two flanking arms of DNA upstream and downstream of the targeted gene. The linear fused PCR amplicon was used directly for transformation into *Synechocystis* by natural transformation. The chloramphenicol resistant transformants were obtained and passed several times on fresh BG11 plates supplemented with 10 μg mL^-1^ chloramphenicol to achieve full chromosome segregation (confirmed by PCR). The mutants and the wild type were grown in the BG11 medium (pH 7.5) in 100-mL flasks each with 25 mL medium, the light intensity was approximately 50 μmol photons m^-2^s^-1^ and the illuminating incubator was 130 rpm, the temperature was controlled at 30°C (HNY-211B Illuminating Shaker, Honour, China). All mutants were first cultivated in BG11 culture with 10 μg/ml chloromycetin for 48 h and then inoculated into BG11 culture without chloromycetin. The growth was determined by cell density measured at OD_630_ on a UV-1750 spectrophotometer (Shimadzu, Japan) every 12 h. For each mutant, three biological replicates were established independently, and each sample was measured in triplicates (Zhu et al., 2015). To confirm the growth patterns, growth experiment of every knockout mutant was repeated at least three times independently, then the growth rates of all mutants were calculated (Supplementary Table S1). Only the growth rates between different RR mutants and wild type with *p*-value <0.005 by *t*-test were considered a significant growth difference.

### LC-MS Based Metabolomics Analysis

Liquid chromatography-mass spectrometry based targeted metabolomics was performed according to the protocol described previously (Wang et al., 2014). All chemicals used for LC-MS metabolomics analysis were obtained from Sigma–Aldrich (Taufkirchen, Germany). For metabolomic analysis, the wild type and the mutant cells were collected at 48 and 72 h, respectively, and each sample was prepared with three biological replicates. Due to the large amount of cultivation needed to finish the comparative experiments of 32 TRs, the samples had to be cultivated, prepared, and analyzed in five batches. A separate cultivation and analysis of the wild type as control was conducted for every batch to minimize possible batch difference. Briefly, the cells were collected by centrifugation at 7500 × *g* for 8 min at 4°C (Eppendorf 5430R, Hamburg, Germany), quenched, and extracted rapidly with 900 μL of 80:20 MeOH/H_2_O (-80°C) and then frozen in liquid nitrogen. The samples were then frozen-thawed three times to release metabolites from the cells. The supernatant was collected after centrifugation at 15,000 × *g* for 5 min at -4°C and then stored at -80°C. The remaining cell pellets were re-suspended in 500 μL of 80:20 MeOH/H_2_O (-80°C), and then the above extraction process was repeated. The supernatant from the second extraction was pooled with that from the first extraction and stored at -80°C until the LC-MS analysis was conducted. LC-MS analysis was conducted on an Agilent 1260 series binary HPLC system (Agilent Technologies, Waldbronn, Germany) using a Synergi Hydro-RP (C18) 150 mm × 2.0 mm ID, 4-μm 80-Å particle column (Phenomenex, Torrance, CA, USA), coupled to an Agilent 6410 triple quadrupole mass analyzer equipped with an electrospray ionization (ESI) source. Data was acquired using Agilent Mass Hunter workstation LC/QQQ acquisition software (version B.04.01), and chromatographic peaks were subsequently integrated via Agilent Qualitative Analysis software (version B.04.00). A total of 24 metabolites were selected for LC-MS-based targeted metabolite analysis in this study. All data of metabolomic profiling was first normalized by the internal control and the cell numbers of the samples.

The 24 targeted metabolites include acetyl coenzyme A (AcCOA), adenosine 5′-diphosphate (ADP), adenosine-5′-diphosphoglucose (ADP-GCS), α-ketoglutaric acid (AKG), adenosine 5′-monophosphate (AMP), adenosine 5′-triphosphate (ATP), coenzyme A hydrate (CoA), dihydroxyacetone phosphate (DHAP), D-fructose 1,6-bisphosphate (FBP), D-fructose 6-phosphate (F6P), sodium fumarate dibasic (FUM), DL-glyceraldehyde 3-phosphate (GAP), D-glucose 6-phosphate (G6P), L-glutamic acid (GLU), α-nicotinamide adenine dinucleotide (NAD), reduced α-nicotinamide adenine dinucleotide (NADH), nicotinamide adenine dinucleotide phosphate (NADP), reduced nicotinamide adenine dinucleotide phosphate (NADPH), uridine 5′-diphosphoglucose (UDP-GCS), oxaloacetic acid (OXA), phosphor (enol)pyruvic acid (PEP), D-(-)-3-phosphoglyceric acid (3PG), D-ribose 5-phosphate (R5P), and D-ribulose1,5-bisphosphate (UDP-GCS), uridine 5′-diphosphoglucose (RiBP).

## Statistical Analysis

The metabolomic profiles were further normalized by comparing relative values of the mutants to the wild type, and then log2 transformed. The data were subjected to Principal Component Analysis (PCA) using software SIMCA-P 11.5 (Laiakis et al., 2010). The PCA analysis is a statistical method to find outliers in the whole set of data. Samples with *p*-value < 0.05 by hotelling t2 statistic were considered significantly different. For Euclidean distance calculation, we used the *dist* function in *R* software after data normalization. Only the distances larger than the upper quartile could be considered as the most affected mutants. Hierarchical clustering analysis was conducted using a *R* software (Deu-Pons et al., 2014).

### TR Family Analysis

Protein sequences of all the 32 TR genes were downloaded from NCBI^1^. To define potential TR families, we used BLAST software for homology identification, only those TR with 80% aligned coverage with *E*-value < 1e-20 were consider as same families.

### Protein–Protein Interaction (PPI) Network Analysis

A protein–protein interaction (PPI) dataset of *Synechocystis* was downloaded from the STRING database (http://www.string-db.org/) (Jensen et al., 2009). STRING aggregates data and predictions stemming from a wide spectrum of cell types and environmental conditions, and aims to represent the union of all possible protein–protein links. In the STRING database, several types of evidence for the association, including genomic context, high-throughput experiments, conserved co-expression and previous biological knowledge were used to calculate a single combined score for each gene in the genome. In this study, only those experimentally validated were applied to construct the PPI network to cover potential protein-protein connections, and the notes of all the proteins in this study were renamed using gene IDs (Szklarczyk et al., 2011).

## Results and Discussion

### Comparative Growth Analysis of TR Mutants

**FIGURE 1:**
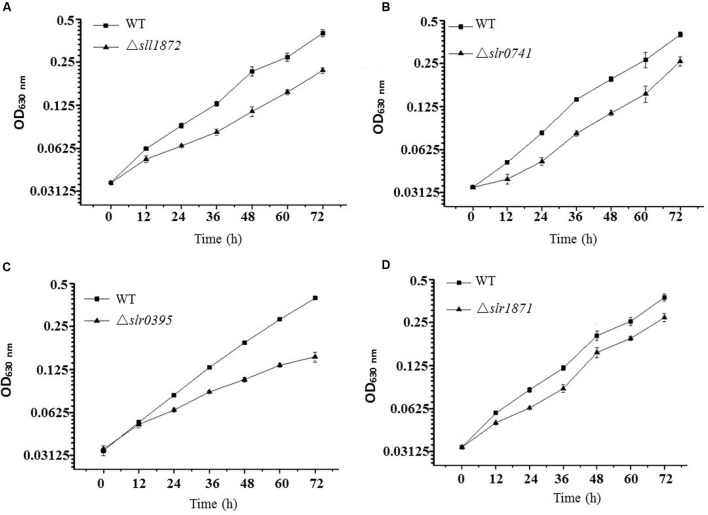
**Growth time courses of the wild type and the mutants in BG11 media.** **(A)** The wild type and the Δ*sll1872* mutant. The block represents the wild type and the triangle represents the Δ*sll1872* mutant. **(B)** The wild type and the Δ*slr0741* mutant. The block represents the wild type and the triangle represents the Δ*slr0741* mutant. **(C)** The wild type and the Δ*slr0395* mutant. The block represents the wild type and the triangle represents the Δ*slr0395* mutant. **(D)** The wild type and the Δ*slr1871* mutant. The block represents the wild type and the triangle represents the Δ*slr1871* mutant.

Although bioinformatics analysis of TRs in *Synechocystis* based on the sequence similarity has been conducted previously (Huffman and Brennan, 2002; Los et al., 2010), their functional classification using experimental approaches is still insufficient. A library of single deletion mutants for 32 TR-encoding genes in *Synechocystis* was constructed and confirmed previously in our laboratory (Zhu et al., 2015), and the majority of them have not been functionally characterized. To seek more functional information for these *Synechocystis* TRs, we first measured differential growth of all TR mutants in normal BG11 medium in flask cultivation, in parallel with the wild type *Synechocystis*. While most of the mutants grew equally well as the wild type (**Supplementary Figure S2**), the comparative analysis showed that four TR mutants, Δ*slr0395*, Δ*slr1871*, Δ*slr0741*, and Δ*sll1872*, grew poorly in the BG11 medium when compared with the wild type (**Figure 1** and Supplementary Table S1), suggesting that the function of these four TRs might be related to key metabolism necessary for normal growth in the BG11 autotrophic growth medium. Among them, deletion of *slr0395* (*ntcB*) caused the most significant growth arrest, with approximately 40% of the growth compared to the wild type after cultivation of 72 h, while deletion of *slr1871* (*pirR*) resulted in the least growth defect, with only 15% of the growth compared to the wild type after 72 h cultivation.

Slr0395 has previously been annotated as nitrite-responsive transcriptional enhancer NtcB in *Synechocystis*, on the basis of the inability of the Δ*slr0395* mutant to rapidly accumulate the transcripts of the nitrate assimilation genes upon induction and to respond to nitrite. In the *ntcB* mutant, activities of the nitrate assimilation enzymes were 40 to 50% of the wild type level, and the cells grew on nitrate at a rate approximately threefold lower than that of the wild type (Aichi et al., 2001).

Slr1871 was previously annotated as PirR of a LysR family, whose encoding gene is located immediately upstream of *pirAB* encoding an ortholog of pirin in the divergent direction, and DNA microarray analysis indicated that PirR repressed expression of closely located ORFs, *slr1870* and *mutS* (*sll1772*), in addition to *pirAB* and *pirR* itself (Hihara et al., 2004). Slr0741 was previously found to encode a negative regulator of the Pi regulon and its insertional inactivation in *Synechocystis* led to increase of the intracellular polyP level (Morohoshi et al., 2002); Slr0741 was also found involved in transduction of the phosphate-limitation signal in *Synechocystis* (Juntarajumnong et al., 2007), and it was also up-regulated upon ethanol stress as revealed by RNA-seq analysis (Wang et al., 2012). Currently no functional information is available for Sll1872.

### Metabolomic Analysis Reveals Functional Diversity of TRs

Liquid chromatography-mass spectrometry-based metabolomics analysis has been recently used to investigate cyanobacterial metabolism due to its advantages toward chemically unstable metabolites, such as the hydrolytically unstable nucleotides (i.e., ATP, GTP, cAMP, and PEP) and the redox active nucleotides (i.e., NADPH, NADP) whose determination could be important in deciphering metabolic responses to genetic or physiological changes. Using a protocol optimized in our previous studies (Wang et al., 2014), a LC-MS-based comparative metabolomics analysis was conducted on all TR mutants and the wild type, with 24 key metabolites involved in central carbon metabolism, cellular energy charge and redox monitored in all samples at 48 and 72 h. The cell samples of 32 TR mutants and the wild type used for LC-MS-based metabolomic analysis were cultivated in BG11 media under autotrophic growth condition and collected at both 48 and 72 h, which were corresponding to earlier and latter exponential phases of cell growth. Each sample was prepared with three biological replicates. As for some, metabolite levels might change during the 8 min centrifugation procedure, a faster centrifugation method may be considered. To reduce the sampling time and maintain the metabolites as much as possible, a higher rotation rate and a lower temperature should be considered. However, with higher rotation rate, more severe damages may occur to cells. A protection agent may be considered in the future. When the rotation rate was higher than 7500 × g, the cells of *Synechocystis* would be easily broken, leading to less metabolites being preserved.

**FIGURE 2:**
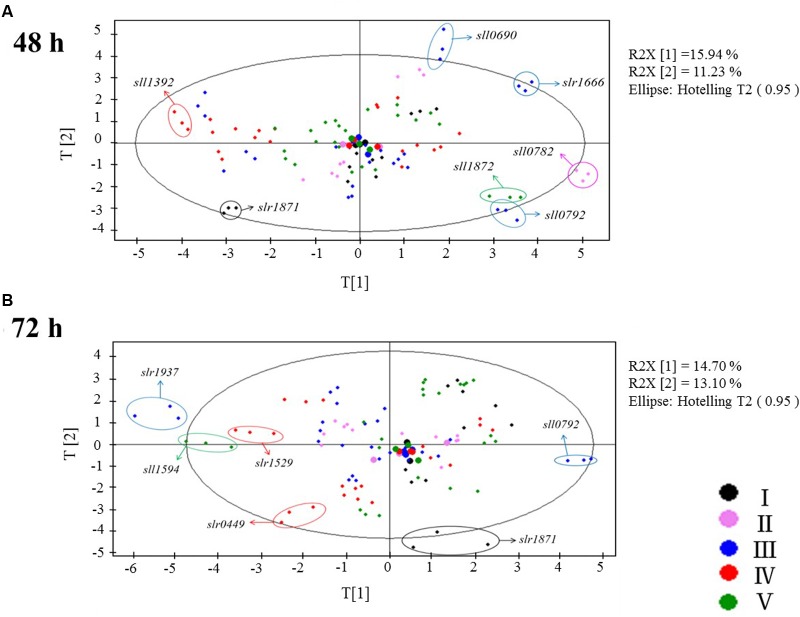
**Targeted LC-MS metabolomic analysis.** **(A)** PCA plots of the LC-MS metabolomic profiles at 48 h. **(B)** PCA plots of the LC-MS metabolomic profiles at 72 h. I, II, III, IV, and V are experiments conducted in five separate times with different colors represent for each batch: black, purple, blue, red, green were for batch I, II, III, IV, V separately.

Several patterns were observed in the PCA plots of the metabolomic data (**Figure 2**): (i) As a large number of cultivation was needed to finish the comparative experiments of 32 TRs, the samples had to be cultivated, prepared and analyzed in five batches. To minimize possible batch difference, a separate cultivation and analysis of the wild type as control was conducted for every batch. In the PCA plots, the big dots of five different colors representing the controls of five batches were found clustered together after data normalization, demonstrating the systematic errors resulting from the experimental design and different cultivation batches were not significant (**Figure 2**); (ii) metabolic profiles of TR mutants were in general well separated at both time points, demonstrating that the LC-MS-based methodology we utilized in this study is sensitive enough to investigate possible differences between controls and all the TR mutants; (iii) except for the four mutants that were grew poorer than the wild type, almost no growth difference was observed between the remaining 28 TR mutants and the wild type when cultivated in the BG11 medium; however, PCA analysis of metabolic profiles showed that these TR mutants were well separated from the wild type in the plots, suggesting that the deletion of these TR-encoding genes has caused significant changes to the cells at the metabolite level. At 48 h, seven TR mutants with the most significant metabolic changes from the wild type control were Δ*slr1871*, Δ*sll1392*, Δ*sll0690*, Δ*slr1666*, Δ*sll1872*, Δ*sll0782*, and Δ*sll0792* (**Figure 2A**, with two components proportions of 15.94 and 11.23%); while at 72 h, six TR mutants, Δ*slr1937*, Δ*sll1594*, Δ*slr1529*, Δ*sll0792*, Δ*slr1871*, and Δ*slr0449* (**Figure 2B**, with two components proportions of 14.70 and 13.10%), displayed the most significant metabolic changes, suggesting a significant functional diversity of TRs in *Synechocystis*, as revealed by the metabolic profiling of selected metabolites related to central carbohydrate metabolism; (iv) only two TR mutants, Δ*sll0792* andΔ*slr1871*, were found significantly regulated at both 48 and 72 h at the metabolite level, suggesting that time- or growth phase-dependent regulation may be involved for most of the responsive TRs; (v) a close examination of the mutant Δ*slr1871* showed that almost all the metabolites involved in central carbohydrate metabolism were down-regulated when compared with the wild type, consistent with the previous results that the *slr1871* (*pirR*) gene had a reduced transcript level during a light-limited linear growth when compared to the exponential growth (Foster et al., 2007). Although it has been reported that the *sll0792* gene encodes ZiaR, a Zn^2+^-responsive repressor of *ziaA* encoding a polypeptide with sequence features of heavy metal transporting P-type ATPases in *Synechocystis* (Thelwell et al., 1998), its regulatory function on cellar metabolism has not yet been established.

To confirm the analysis with PCA, another approach, the *Euclidean* distances calculated based on the different metabolite profiles between each mutant and its control (the wild type), were also determined. In **Supplementary Figure S1**, the TR mutants were ordered based on the degree of metabolic changes when compared with the wild type in descending order. The analysis was conducted separately with metabolomic profiling data of two time points (i.e., 48 and 72 h), and showed that the top changed mutants were Δ*sll0690*, Δ*slr1871*, Δ*sll1670*, Δ*slr1666*, Δ*slr1245*, Δ*sll1957*, Δ*sll1594*, and Δ*sll1872* at 48 h; Δ*slr0449*, Δ*slr1871*, Δ*ssl0564*, Δ*sll1937*, Δ*slr1666*, Δ*slr0115*, Δ*slr0724*, and Δ*sll0690* at 72 h, respectively. The results were consistent with those of the PCA analysis.

### Metabolomic Basis for the Differential Growth in Four TR Mutants

**FIGURE 3:**
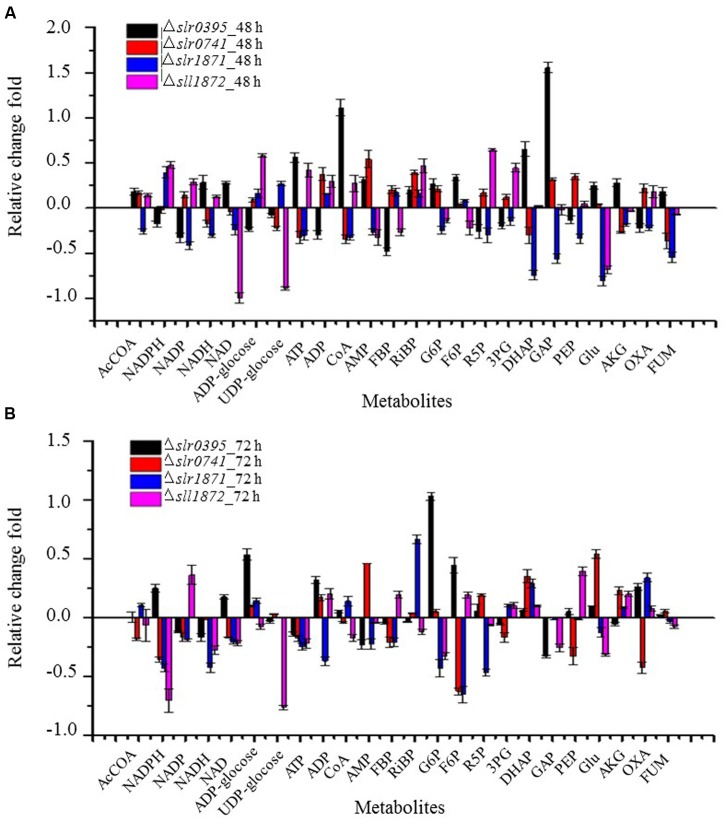
**A detailed analysis of the metabolite abundance in four mutants with differential growth.** **(A)** calculated based on the metabolite profiles of 48 h. **(B)** calculated based on the metabolite profiles of 72 h.

Comparative growth analysis showed that four TR mutants were grown poorly in BG11 medium when compared with the wild type. A detailed analysis of the metabolite abundance of the 24 metabolites in these mutants was then conducted (**Figure 3**). Slr0395 is involved in regulation of nitrate assimilation gene (*ntcB*) (Burnap et al., 2015), so it is expected that the deletion of the *slr0395* (*ntcB*) gene would decrease nitrogen metabolism. Accordingly, the metabolomics analysis showed Glu was up-regulated by 24.2, and 11.7% at 48 h and 72 h, respectively. In addition, abundance of CoA in the Δ*slr0395* was found increased.

Slr0395 and Slr1871 are LysR-type transcriptional regulator protein (LTTRs) that have been found important for regulation of the carbon concentration mechanism (CCM) in cyanobacteria (Daley et al., 2012). In Calvin–Benson cycle, F6P and G3P were converted to GAP, R5P, and then Ru5P and RiBP for CO_2_ fixation (Wang et al., 2011). The metabolomic analysis showed that metabolite RiBP, F6P, GAP, and R5P associated with RiBP were down-regulated in the Δ*slr0395* and Δ*slr1871* mutants (**Figure 3**), which could be responsible for the slow growth in the mutants.

Regulatory function of gene *sll1872* (*lytR*) has not been determined previously. According to our metabolomic analysis, several metabolites related to energy metabolism, including NADP, NADH, NAD and ATP were all down-regulated at both sampling time points, suggesting that the absence of the *sll1872* (*lytR*) may negatively affect the energy metabolism and then reduced the growth of Δ*sll1872* mutant (**Figure 3**).

Interestingly, although the growth of Δ*slr0741* was clearly slower than the wild type, the metabolomic analysis showed no obvious difference at the metabolite level between the Δ*slr0741* mutants and the wild type, implying that its regulatory function may be not directly related to the central carbohydrate metabolism.

### Functional Conservation of TRs

**FIGURE 4:**
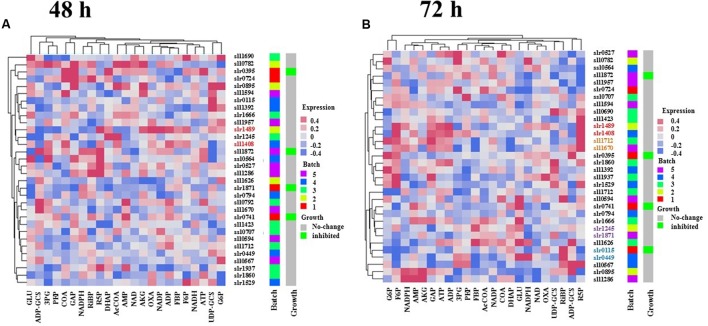
**Comparison of hierarchical clustering trees of metabolomic data.** **(A)** Hierarchical clustering tree based on the metabolite profiles of 48 h. **(B)** Hierarchical clustering tree based on the metabolite profiles of 72 h. The same colored text for some of the gene names represents they were clustered in metabolic level and shared common target proteins.

The finding that a range of metabolic changes occurred in single-deletion TR mutants led to several immediate questions. First, whether the TR mutants with similar metabolic profiles have a close relationship on the evolutionary tree for their encoding genes? Second, whether the TR-encoding genes with a close evolutionary relationship have similar metabolic changes to the gene deletion? Answers to these questions could provide clues to possible function, evolution and origin of the TR genes. To seek answers to the questions, a gene families’ analysis was conducted using full protein sequences of 32 TR genes. The homology analysis showed that 32 TR genes were classified into several different families, indicating different origin during the evolution of TR genes (**Figure 4** and Supplementary Table S3). Meanwhile, several pairs of TRs were found in the same gene family, suggesting possible events of gene duplication in recent evolutionary courses. Interestingly, while comparing the trees resulted from the hierarchical clustering analysis of metabolomic data and the gene families, we found that several pairs of TRs were clustered together in same gene family and the relationship tree generated using metabolomic profiles, suggesting possible functional conservation of the genes during genome evolution (**Figure 4**). At 48 h, one pair of TR genes, *slr1489* (*pchR*) and *sll1408* (*pcrR*), with similar function and evolution was identified; however, when metabolic profiles of 72 h were used, five pairs of TRs were identified. The pairs were *slr0895* (*prqR*) and *sll1286. slr1489* (*pchR*) and *sll1408* (*pcrR*); *sll1712* and *sll1670* (*hrcA*); *slr0115* (*rpaA*) and *slr0449* (*dnr*); *slr1871* (*pirR*) and *slr1245*. Only one pair of TRs, *slr1489* (*pchR*) and *sll1408* (*pcrR*), was identified when using metabolomic profiling data of both time points, suggesting a very conserved role that they might be playing. The difference between 48 and 72 h was probably due to the phase-dependent regulation of TRs in *Synechocystis*, which has been commonly reported in various microbes (Zhu et al., 2015).

Meanwhile, the results also showed a total of 17 pairs of TRs that were clustered together in the metabolomic trees but did not locate in the same gene family (**Figure 4**). Although experimental measurements of more metabolites are still necessary, the preliminary analysis pointed to the possibility of functional convergence of TR genes during the genome evolution, as the results showed that deletion of TR genes of different evolutionary origins caused similar metabolic responses in the mutant cells. For example, the *slr0395* (*ntcB*) and *slr0724* (*sohA*) genes were clustered together in the tree resulted from metabolic profiles of 48 h, deletion of these two TR genes caused CoA and GAP were down-regulation while ADP and ADP-glucose were up-regulated, although they belong to different gene families.

### Functional Inference of TRs

Experimental and computational data from genome-scale PPI analysis has contributed significantly to the understanding of the gene function (Marcotte et al., 1999; Ikeuchi and Tabata, 2001; Sato et al., 2007). In this study, an attempt was also made to apply PPI network of *Synechocystis* to determine possible gene targets of the five pairs of TRs identified above with similar clustering patterns in metabolomic profiles and matching gene family membership. As TRs of each pair have similar evolutionary and metabolomics patterns, it was expected that they might function through the same targets. Based on this hypothesis, we then implemented a strategy to first determine regulatory targets of each TR using PPI network analysis, and then identify the common target genes for every pair of TRs. The analysis allowed inference of possible regulatory gene targets for four out of five pairs of TRs, although no common target gene was identified for the *slr1871* (*pirR*) and *slr1245* pair (Supplementary Table S2).

(i) *sll1408* (*pcrR*) and *slr1489* (*pchR*): *sll1408* is a gene encoding a regulator protein PcrR and repressed by cold sensor Hik33 in *Synechocystis* (Suzuki et al., 2001). A previous study found that *sll1408* and *slr1489* were regulated by iron (and cadmium) and hydrogen peroxide stresses under control of the Slr1738 regulator (Houot et al., 2007). The two mutants did not show growth difference. Our analysis showed that Sll1408 and Slr1489 had four common possible target proteins: Slr0707, Slr0664, Sll0043, and Slr0594, among which Slr0707 is DNA polymerase I involved in the biological process of DNA replication (Sato et al., 2003), *slr0664* encodes a toxin–antitoxin (TA) system belonging to *rel* family (Ning et al., 2011), *sll0043* encodes a protein related to phototaxis (Shin et al., 2008), and *slr0594* was a putative membrane-spanning component of ABC transporters (Wipat et al., 1998), respectively.
(ii) *slr0895* (*prqR*) and *sll1286*: Previous studies showed that the gene *sll1286* was down-regulated by acid stress and sorbitol stress (Ohta et al., 2005; Shapiguzov et al., 2005), while *slr0895* (*prqR*) was found to be an auto-repressor regulating the adaptive responses to the oxidative stress (Cheng and He, 2014). In addition, *sll1286* and *slr0895* (*prqR*) were both encoding regulators of TetR family. As one TetR family regulator protein PfsR has been found as a key regulator of iron homeostasis in *Synechocystis*, and the PrqR (Slr0895) was an auto-repressor regulating the adaptive responses to oxidative stress (Cheng and He, 2014). No growth difference was found between these two mutants. Our analysis showed that Slr0895 and Sll1286 had two common possible target proteins: Slr1705 and Sll0782. *slr1705* (*aspA*) encodes aspartoacylase (Osanai et al., 2014), while *sll0782* is annotated as a helix–turn–helix DNA binding motif without any known function (Zhang et al., 1998). Based on COG and KEGG annotation, Slr1705 may be participating in amino acid transport and metabolism, such as alanine, aspartate and glutamate, which is consistent with the down-regulation of Glu at 72 h in the deletion mutants of *slr0895* (*prqR*) and *sll1286*.
(iii) *sll1670* (*hrcA*) and *sll1712. sll1670* gene encodes an ortholog of HrcA, a negative regulator of heat stress genes (Zorina et al., 2011). Sll1712 is a DNA binding protein (Chen et al., 2014) that was positively regulated by cadmium (Houot et al., 2007). The two mutants did not show growth difference. Our analysis showed that Sll1670 and Sll1712 had two common target proteins: Slr0701 and Sll0794. Slr0701 is a mercuric resistance operon regulator (Hirosawa et al., 1997), *sll0794* (*corR*) encoding a sensor gene involved in Ni^2+^, Co^2+^, and Zn^2+^ sensing and tolerance (García-Domínguez et al., 2000; Mehta et al., 2014), and tolerance to ethanol (Huertas et al., 2014).
(iv) *slr0115* (*rpaA*) and *slr0449* (*dnr*): *slr0449* (*dnr*) encodes a TR belonging to the Crp/Fnr family, which has been found regulated by AbrB2 (Leplat et al., 2013). Slr0115 is related to energy transfer from phycobilisomes to photosystems (Hanke et al., 2011), and deletion of *slr0115* (*rpaA*) resulted in increased efficiency of energy transfer from phycobilisomes to photosystem II relative to photosystem I (Ashby and Mullineaux, 1999). No growth difference was found between these two mutants. Our analysis showed that Slr0115 and Slr0449 had four common target proteins: Sll1196, Sll0745, Slr0884, and Sll1342, among which *sll1196* (*pfkA*) and *sll0745* (*pfkA*) encode two phosphofructokinases (Osanai et al., 2005), and participate in carbohydrate transport and metabolism, *slr0884* (*gap1*) and *sll1196* (*pfkA*) showed similar enhancement of expression through overexpression of *rre37* (*sll1330*) (Okada et al., 2015), while *sll1342* (*gap2*) encodes glyceraldehyde-3-phosphate dehydrogenase whose pathway involves in F6P, GAP, and R5P (Rowland et al., 2011; Lee et al., 2015). As a phosphofructokinase encoding gene, the expression of *sll1196* (*pfkA*) could affect the accumulation of F6P (Tabei et al., 2007), while the components GAP and FBP that were related to F6P phosphorylation were also changed.

In this study, 32 knockout mutants for putative TR-encoding genes of *Synechocystis* were constructed and comparatively analyzed via LC-MS-based metabolomics. Four mutants, *sll1872 (lytR). slr0741 (phoU). slr0395 (ntcB)*, and *slr1871 (pirR)*, showed differential growth patterns in BG11 medium when compared with the wild type. In the remaining TR mutants that did not show growth difference compared with the wild type, metabolomic profiling showed that they were clearly different at the metabolite level, suggesting significant functional diversity of TRs in *Synechocystis*. Finally, protein-protein interaction network analysis predicted possible regulatory targets of TRs.

## Author Contributions

LC and WZ conceived and designed the study. MS and XZ performed the experiments. MS, XZ, GP, LC, and WZ analyzed the data and wrote the manuscript. All authors read and approved the manuscript.

## Conflict of Interest Statement

The authors declare that the research was conducted in the absence of any commercial or financial relationships that could be construed as a potential conflict of interest.

## Footnotes

1 http://www.ncbi.nlm.nih.gov

## Supplementary Material

**FIGURE S1 | Euclidean distances between each mutant and its control.** **(A)** Calculated based on the metabolite profiles of 48 h; **(B)** Calculated based on the metabolite profiles of 72 h.

**FIGURE S2 | Growth curves of 28 mutants without different growth with the wild type.** The black block represents for the wild type and the red dot represents for corresponding mutant.
