# Supplementary figures and images for: Functional Diversity of Transcriptional Regulators in the Cyanobacterium Synechocystis sp. PCC 6803
Source: Front Microbiol. 2017 Feb 21;8:280. doi: 10.3389/fmicb.2017.00280 (PMC5318462; doi:10.3389/fmicb.2017.00280)

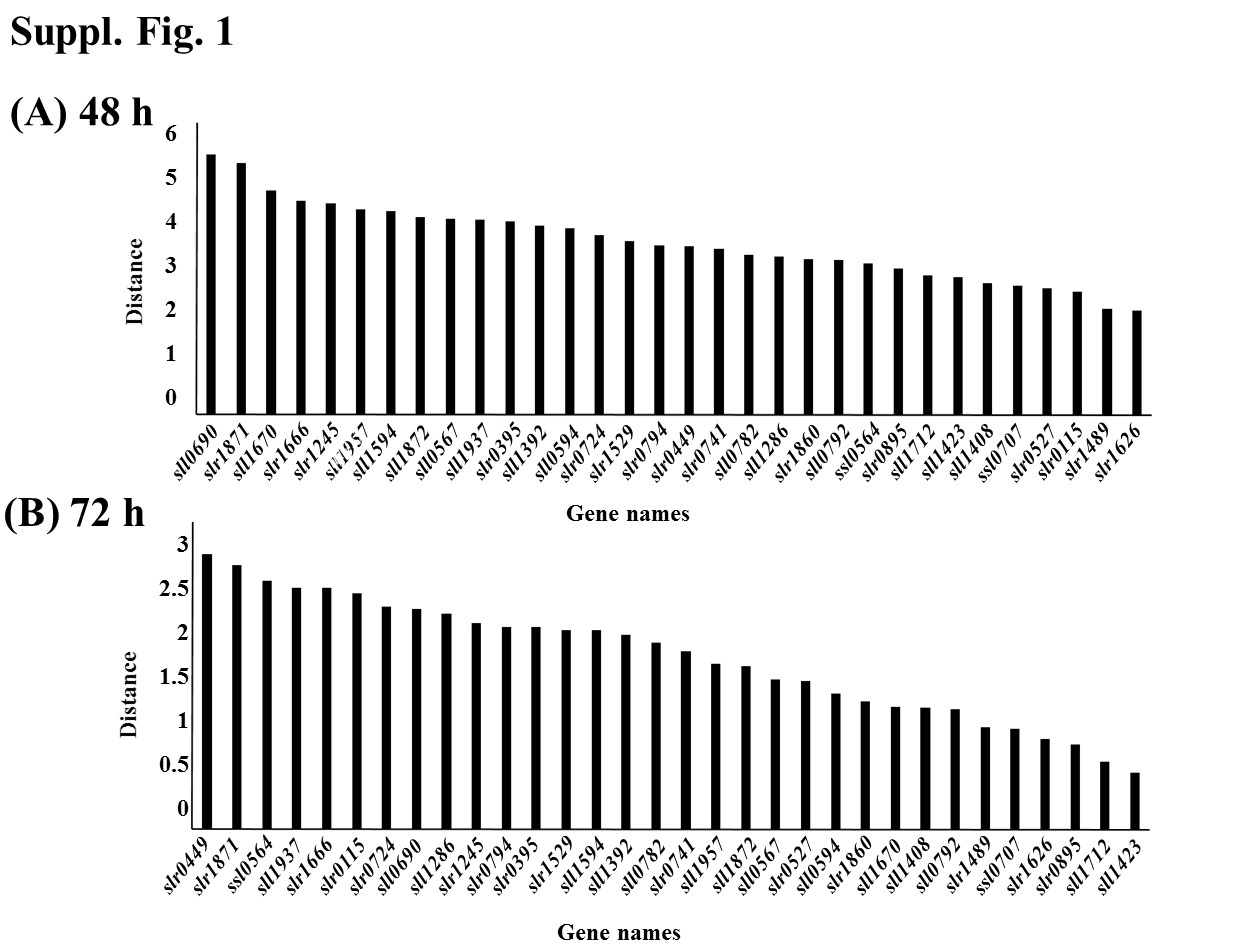

Supplement: Supplementary file 1 [file Image_1.JPEG]

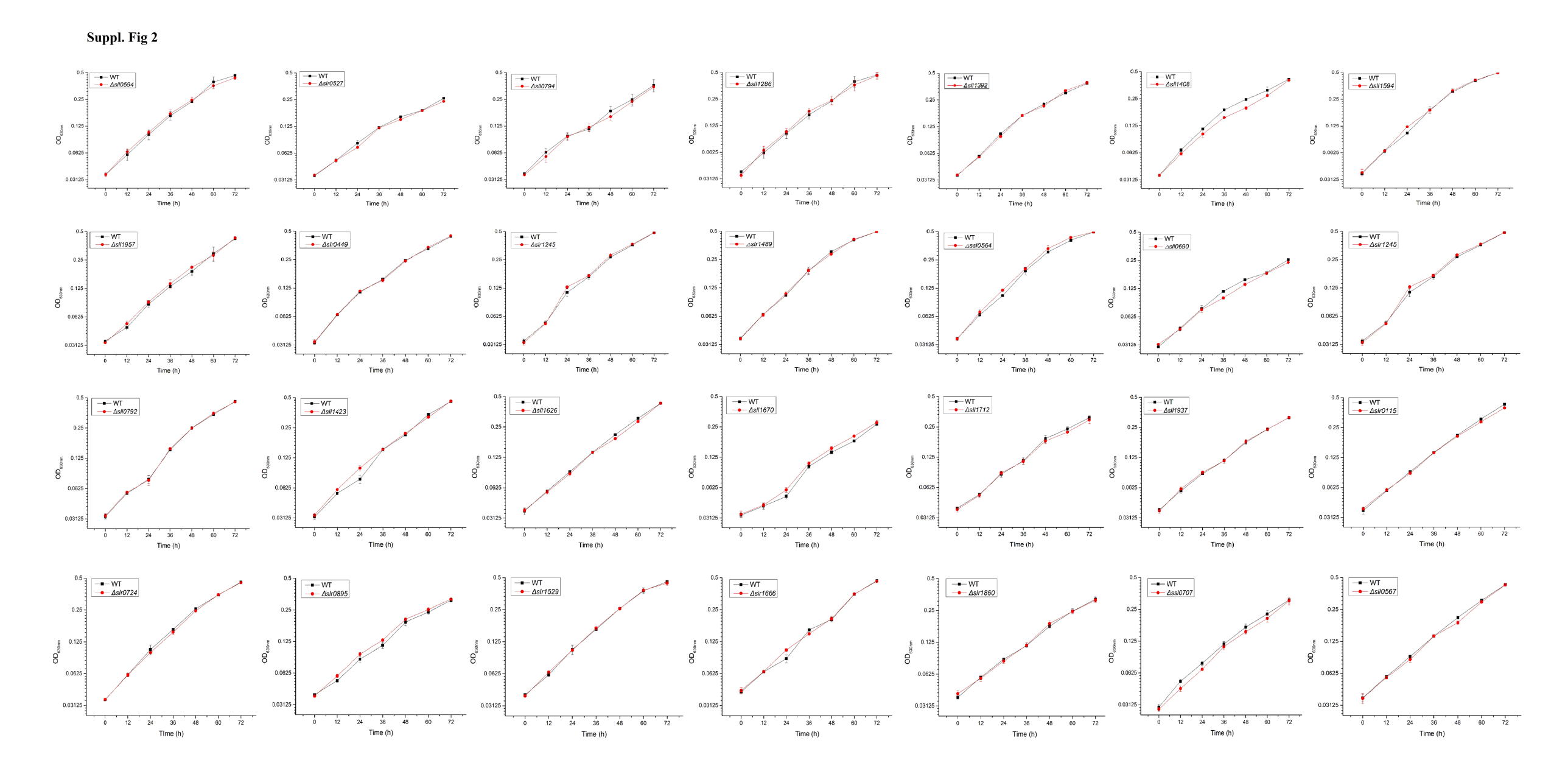

Supplement: Supplementary file 2 [file Image_2.JPEG]
